# Using Video Cameras to Assess Physical Activity and Other Well-Being Behaviors in Urban Environments: Feasibility, Reliability, and Participant Reactivity Studies

**DOI:** 10.2196/66049

**Published:** 2024-12-16

**Authors:** Jack S Benton, James Evans, Jamie Anderson, David P French

**Affiliations:** 1 Manchester Centre for Health Psychology Division of Psychology and Mental Health The University of Manchester Manchester United Kingdom; 2 Manchester Urban Institute Department of Geography The University of Manchester Manchester United Kingdom

**Keywords:** unobtrusive observation, video cameras, measurement, physical activity, well-being, urban environments

## Abstract

**Background:**

Unobtrusive observation is a promising method for assessing physical activity and other well-being behaviors (eg, social interactions) in urban environments, without participant burden and biases associated with self-report. However, current methods require multiple in-person observers. Using video cameras instead could allow for more accurate observations at lower cost and with greater flexibility in scheduling.

**Objective:**

This research aimed to test the feasibility of using stationary wireless video cameras to observe physical activity and other well-being behaviors, and to assess its reliability and potential participant reactivity.

**Methods:**

Across 3 cross-sectional studies, 148 hours of video recordings were collected from 6 outdoor public spaces in Manchester, United Kingdom. The videos were coded by 3 researchers using MOHAWk (Method for Observing Physical Activity and Wellbeing)—a validated in-person observation tool for assessing physical activity, social interactions, and people taking notice of the environment. Inter- and intrarater reliabilities were assessed using intraclass correlation coefficients (ICCs). Intercept surveys were conducted to assess public awareness of the cameras and whether they altered their behavior due to the presence of cameras.

**Results:**

The 148 hours of video recordings were coded in 85 hours. Interrater reliability between independent coders was mostly “excellent” (ICCs>0.90; n=36), with a small number of “good” (ICCs>0.75; n=2), “moderate” (ICCs=0.5-0.75; n=3), or “poor” (ICCs<0.5; n=1) ICC values. Reliability decreased at night, particularly for coding ethnic group and social interactions, but remained mostly “excellent” or “good.” Intrarater reliability within a single coder after a 2-week interval was “excellent” for all but 1 code, with 1 “good” ICC value for assessing vigorous physical activity, indicating that the coder could reproduce similar results over time. Intrarater reliability was generally similar during the day and night, apart from ICC values for coding ethnic group, which reduced from “excellent” to “good” at night. Intercept surveys with 86 public space users found that only 5 (5.8%) participants noticed the cameras used for this study. Importantly, all 5 said that they did not alter their behavior as a result of noticing these cameras, therefore, indicating no evidence of reactivity.

**Conclusions:**

Camera-based observation methods are more reliable than in-person observations and do not produce participant reactivity often associated with self-report methods. This method requires less time for data collection and coding, while allowing for safe nighttime observation without the risk to research staff. This research is a significant first step in demonstrating the potential for camera-based methods to improve natural experimental studies of real-world environmental interventions. It also provides a rigorous foundation for developing more scalable automated computer vision algorithms for assessing human behaviors.

## Introduction

Characteristics of urban environments, such as green infrastructure, street design, and land use mix, can influence physical activity [1] and other “well-being behaviors,” including social interactions [2] and engagement with nature [3]. However, robust evidence on the most effective environmental interventions to promote these well-being behaviors is scarce [4].

A fundamental weakness of the evidence base is a reliance on self-report measures, most commonly surveys, which are susceptible to issues such as recall bias [5], measurement reactivity [6], and low response rates [7]. For example, survey response rates as low as 14% are not uncommon in physical activity research in this area [7], increasing the risk of selection bias. Another commonly used method involves infrared sensors, which offer a low-cost solution for monitoring footfall over long periods of time. However, these sensors cannot capture details on the frequency, intensity, duration, and type of physical activity, nor can they assess other behaviors relevant to the use of urban environments. Further, they are prone to errors, such as systematically undercounting groups of users as single users [8].

Systematic observation (ie, direct observations of behavior using predetermined criteria) is a promising alternative method for assessing human behavior in urban environments [9]. Observations can be unobtrusive, where participants are not aware they are being assessed, and thus can be carried out without participant burden and selection or reactivity biases typically associated with self-report measures. Several systematic observation tools are now available, predominantly for assessing physical activity in community and recreational settings [10-17]. One of the key applications of these tools is in before and after studies of natural experiments (ie, “real-world” interventions) to evaluate the impact of environmental changes, such as park improvements [18], urban greenways [19], new walking infrastructure [20], and active neighborhood projects [21]. Due to the impracticality of conducting randomized controlled trials in these contexts, natural experiments provide valuable opportunities for assessing the effects of environmental interventions on health behaviors.

However, efforts to use observation tools are impeded by the fact that considerable time and cost is needed to deploy multiple observers to conduct “live” in-person observations. It is, therefore, expensive and impractical to conduct the number of observations necessary to detect meaningful effect sizes and capture the effects of environmental interventions across multiple locations and time points in natural experimental studies. Further, relying on in-person observers limits the ability to conduct observations outside of the traditional working hours, such as during evenings and weekends. Conducting observations at night is also impractical due to safety concerns for the observers. For these reasons, behavior observation methods have failed to accumulate a high-quality, intervention-based evidence base, despite being advocated in seminal works for over half a century [13,22-25].

The development of video camera–based observation methods could address these issues. Specifically, video cameras can be used to collect recordings in public spaces, which can then be watched and coded by researchers. Coding video recordings that can be paused and replayed, rather than conducting live in-person observations, may enhance the accuracy of observations and allow a broader range of behaviors to be assessed. Replacing in-person observers with video cameras would offer greater flexibility in the frequency and scheduling of observations, reduce the risks associated with researchers working alone in public spaces for extended periods, and enable nighttime observations. Further, video cameras can be deployed at a relatively low cost, and fewer researchers are required for data collection and coding, making it easier and less expensive to conduct natural experimental studies of environmental interventions.

A key barrier to using video cameras in public spaces is the ethical and information governance challenges associated with the recording of identifiable images of participants, which constitutes personal data. Stringent data protection laws have discouraged many researchers from adopting camera-based observation methods. This issue is particularly relevant for researchers in the European Union, where the General Data Protection Regulation (GDPR) introduced in 2018 serves as a leading global standard for data protection. Despite these challenges, guidelines have been developed on how to conduct camera-based research ethically and in compliance with data protection laws, addressing issues related to data protection, privacy, informed consent, and confidentiality [26]. Ethical guidelines also exist for the use of wearable cameras in health behavior research [27,28] and for visual research more generally [29,30]. In a related area of research, guidelines have been established for the legal analysis of street view imagery [31]. The use of street view imagery, such as Google Street View, has had transformative impacts on the assessment of microscale environmental features related to physical activity [32].

Despite this progress, only a few studies have used camera-based observation methods to assess physical activity behavior in outdoor environments. Much of the research to date has relied on still images [33,34], which provide only limited snapshots of behavior. The small amount of video-based research has predominantly used preexisting video cameras (eg, outdoor webcams [35]), which restrict opportunities to evaluate sites that are not already covered by those cameras. More recently, researchers have started to code video recordings from drones [36-38], offering more flexibility around site selection. However, drones still require a trained drone pilot to be present during observations to operate the flight path, and drones cannot be used in poor weather or at night. There are also safety concerns from drone crashes and public disturbance from trespassing over private land, leading many governments to implement stricter regulations on drone use [39].

An alternative approach that addresses these limitations is the use of stationary video cameras that can be temporarily installed in public spaces. Recent advances in video technology have made it possible for publicly available wireless video cameras to provide sufficient battery life and image resolution for research purposes, even for those with limited videography skills. A recent study in the United States deployed stationary overhead cameras to assess the total number of people and number of people physically active in video recordings [40]. However, it remains unclear whether the deployment of wireless video cameras in public spaces can reliably and validly assess physical activity and other well-being behaviors, as well as equity-related variables such as age, gender, and ethnic group.

There are several indicators of reliability relevant to a camera-based observation tool, 2 of which are interrater reliability (the degree of agreement among independent coders) and intrarater reliability (the consistency of a single coder’s measurements over time). It is particularly important to test reliability at night, when video images may be less clear, potentially reducing coding reliability. Another methodological concern is whether deploying cameras in public spaces may inadvertently introduce bias due to participant reactivity, where individuals become aware of being recorded and subsequently alter their behavior.

To address these methodological concerns, this paper reports 3 studies that aimed to test the feasibility of conducting reliable and nonreactive systematic observation of physical activity and other well-being behaviors by coding video recordings collected from stationary wireless video cameras. Specific objectives of these three studies were to (1) test the feasibility of deploying wireless video cameras as a research tool in public spaces (studies 1, 2, and 3); (2) assess interrater reliability between pairs of coders (study 3); (3) assess intrarater reliability within a single coder at 2 separate intervals (study 3); (4) compare inter- and intrarater reliability between day and night (study 3); and (5) use intercept surveys to examine public space users’ awareness of and reactivity to cameras (study 3).

## Methods

### Study Design

There were 3 prospective cross-sectional studies. Each study had 2 phases of camera-based data collection. The first phase involved ensuring that video recordings were taken without objections from members of the public, testing the clarity of images, and determining the optimal positioning of cameras. Any adjustments were made before proceeding to the second phase of data collection that was used for coding. In the third study, intercept surveys were also conducted with public space users to examine their awareness of and reactivity to our cameras. These studies are reported in accordance with the Strengthening the Reporting of Observational Studies in Epidemiology (STROBE) cross-sectional reporting guidelines [41] (Multimedia Appendix 1).

### Setting

All 3 studies were set in outdoor public spaces in Manchester, United Kingdom—a densely populated city in North West England with a population of 555,741 [42]. Studies 1 and 3 were set on the main University of Manchester campus, which is located a mile south of Manchester city center. Study 2 was set in Levenshulme, which is a highly deprived urban neighborhood in South Manchester. The Levenshulme ward has an index of multiple deprivation score of 39.73 [43], belonging to the most deprived index of multiple deprivation quintile (≥34.18) [44]. All sites were chosen as they were already being monitored by existing closed-circuit television (CCTV) cameras for security purposes and were, therefore, perceived as lower risk sites to the university ethics committee.

Each study took place at different times of the year—study 1 in autumn (November 2020), study 2 in spring (March 2021), and study 3 in summer (June 2022). Weather conditions were clement, with no precipitation observed in any video recordings.

### Measures and Equipment

#### Systematic Observation Tool: MOHAWk

Video recordings were coded using MOHAWk (Method for Observing Physical Activity and Wellbeing)—a reliable and valid in-person observation tool for assessing physical activity and 2 other well-being behaviors (connect—social interactions and take notice—taking notice of the environment) in urban environments [11]. There is evidence of high interrater reliability between pairs of in-person observers when using MOHAWk and evidence of criterion-related validity [11]. The physical activity codes used in MOHAWk (sedentary, walking, and vigorous) are based on previous observation tools that have been validated using heart rate monitors [45], pedometers [46], and accelerometers [47].

MOHAWk was chosen over other observation tools, such as the System for Observing Play and Active Recreation in Communities (SOPARC) [14], because (1) MOHAWk assesses additional behaviors that are important for well-being beyond physical activity; (2) it uses continuous scanning to count all individuals and their activities during the 1-hour observation periods, rather than relying on brief observational scans; and (3) it has been validated for use in the United Kingdom. To date, MOHAWk has been used in at least 5 natural experimental studies of urban environment interventions [20,48-51].

#### Video Camera Equipment

**Figure 1:**
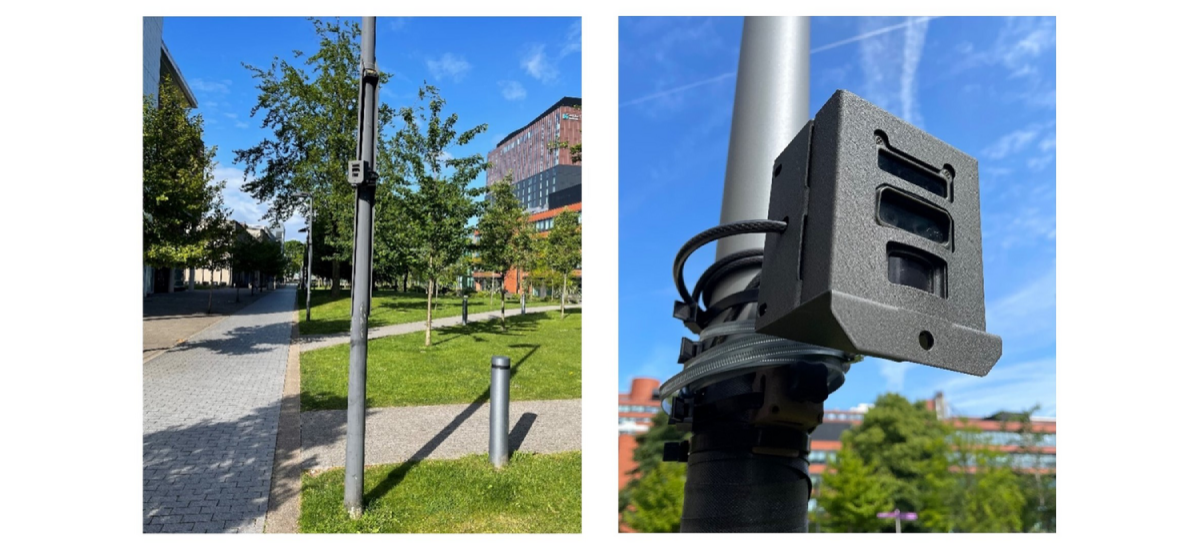
Photographs of a mounted camera. Photographs taken by JSB at site 3B.

We used “Reconyx XS8 UltraFire” wireless video cameras (Figure 1), chosen for their suitability based on several factors, including size, image quality or resolution, battery life, memory capacity, password protection, availability, and cost. Each camera was configured to capture color video at 720 pixels and 30 frames per second, recording videos at preset time intervals regardless of motion detection. Nighttime images were captured using an infrared flash. The cameras were powered by 12 AA batteries, which require replacement after approximately 24 hours of continuous recording. Although compact solar panels and power banks are compatible with these cameras to extend battery life and deployment time, they were not used in these studies.

Each camera is password-protected and supports up to 256 GB of memory on a removable secure digital card. For security, the cameras were placed inside locked security cases and mounted to lampposts approximately 2.5 meters above the ground using steel jubilee clips, cable ties, and a cable lock. As of October 2020, the total 1-off cost for each camera and additional equipment was approximately £758 (≈US $979), excluding Value Added Tax.

#### Privacy Notice Signs

Multiple A3 privacy notice signs were displayed at all study sites when videos were being recorded. A more detailed participant information sheet was displayed as a paper print-out underneath each sign, which also included a QR code to a web-based version. Displaying privacy notices is not only in line with the GDPR but is also recommended by other existing guidance for using video surveillance in public spaces, such as the UK Government Surveillance Camera Code of Practice [52].

### Procedures

#### Overview of MOHAWk

Coders used the MOHAWk tool to assess the following estimated information for each person that entered a predetermined target area during prespecified hour-long observation periods—gender (female or male), age group (infant, child, teen, adult, or older adult), ethnic group (White or non-White), physical activity level (sedentary, walking, or vigorous), social interaction (connect or no connect), and taking notice of the environment (take notice or no take notice). “Connect” behaviors occur when individuals are engaging or interacting with a person or the people around (eg, talking, holding hands, and group activity). “Take Notice” behaviors occur when individuals stop or slow down and appear as if they are making a conscious decision to appreciate their surroundings (eg, taking a photograph, engaging with wildlife, and extended viewing of a scene).

In accordance with MOHAWk procedures, the unit of coding is the behavior, meaning that the number of individuals performing each behavior is counted within each hour-long observation period. Therefore, the same person can be recorded as participating in multiple behaviors. However, each behavior cannot be recorded more than once for the same individual within the same observation period. For example, if a person speaks to someone and then hugs someone else during the same observation period, this would be recorded as one “Connect” behavior for that person. To replicate in-person MOHAWk observation procedures, coders attempted to avoid double counting the same person within each hour-long observation period.

Data were separated into 5-minute blocks within each observation period to provide a bigger sample size for inter- and intrarater analyses. Each coder recorded the total time spent on coding, which was then compared to the total hours of video recordings they coded. Table 1 summarizes the frequency and scheduling of coding for each study.

**Table 1:** Summary of coding for each study.

Study	Site	Videos coded	Coder (videos coded)	Coding schedule^a^	
				Dates	Times
1	1	8 hours	Coder 1 (8 hours)	Wednesday November 25, 2020 Thursday November 26, 2020	Noon-4 PM Noon-4 PM
2	2	12 hours	Coder 1 (12 hours)	Thursday March 18, 2021 Friday March 19, 2021	Noon-4 PM 8 AM-4 PM
3	3A 3B 3C 3D	20 hours 20 hours 20 hours 20 hours	Coder 2 (80 hours), Coder 1 (8 hours), and Coder 3 (40 hours^b^)	Tuesday June 14, 2022 Friday June 17, 2022 Wednesday June 15, 2022 Saturday June 18, 2022	8 AM-9 AM, noon-1 PM, 5 PM-6 PM, 7 PM-8 PM, 8 PM-9 PM, 10 PM-11 PM, and 11 PM-midnight Midnight-1 AM, 1 AM-2 AM, and 2 AM-3 AM

^a^This does not include any video recordings that were collected but were not coded (eg, video recordings for study 3 were collected between 8 AM and 9 PM).

^b^Includes the additional 20 hours of videos that were recoded for intrarater reliability purposes.

#### Study 1

**Figure 2:**
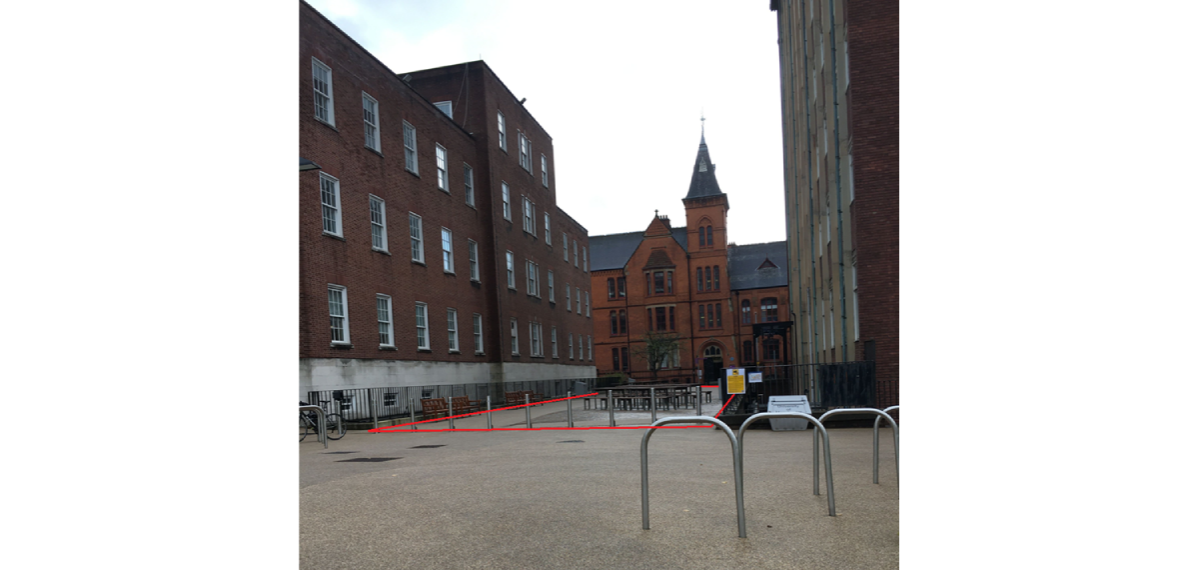
Photograph of site 1 and approximate target area boundary (in red) for study 1. Photograph taken by JSB in October 2020.

Study 1 was conducted at 1 outdoor site on the University of Manchester campus (Figure 2). This location was chosen as it was perceived to be safe for piloting these methods because of 24 hours-a-day lighting, CCTV, and the presence of university security staff. One camera was mounted, and 8 hours of video recordings were captured over 2 days (Table 1). Previous research has shown that shortened observation schedules (eg, 2 days, 4 times a day) can yield reliable estimates of activity in a public space [11,53]. The lead author (JSB) coded all 8 hours of video recordings. JSB has considerable expertise in using MOHAWk, having conducted over 300 hours of in-person observations.

#### Study 2

**Figure 3:**
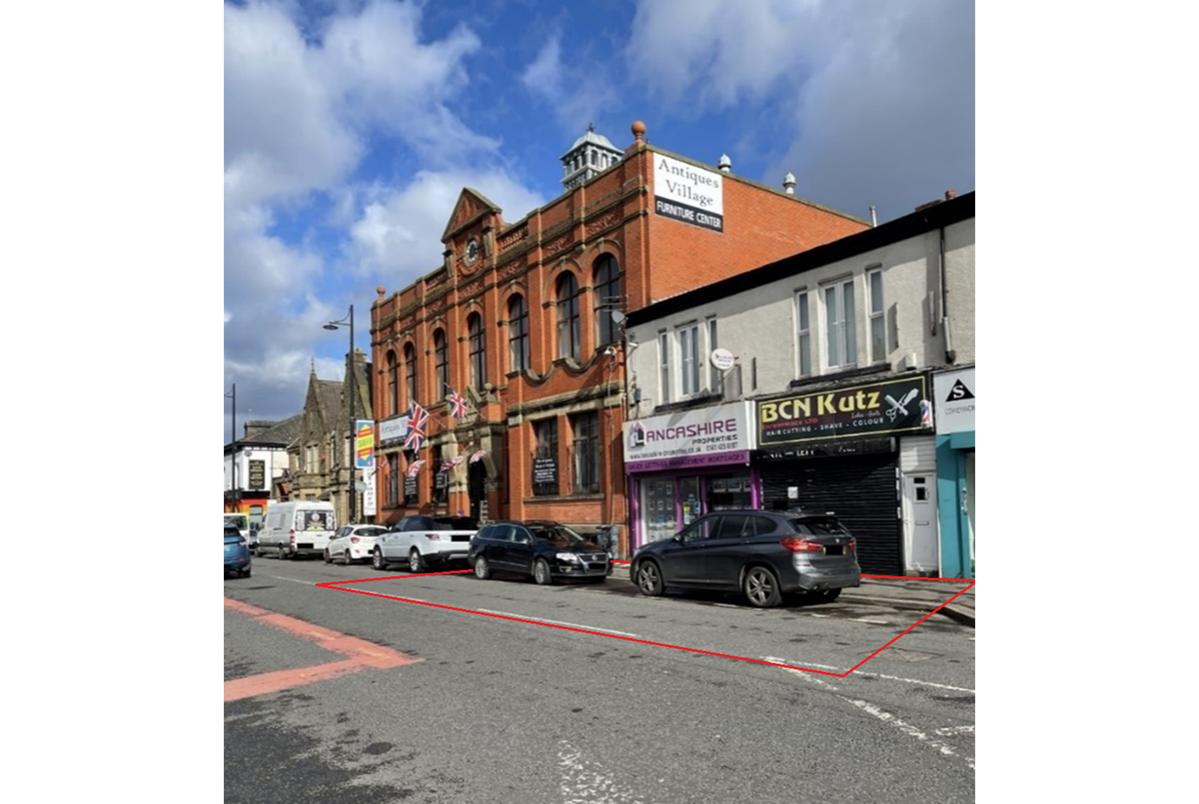
Photograph of site 2 and approximate target area boundary (in red) for study 2. Photograph taken by JSB in March 2021. All identifying features have been masked.

Study 2 aimed to test the feasibility of using 2 cameras mounted at different angles to provide improved visual coverage of the target area. A high street in Levenshulme was selected for this study (Figure 3) due to existing relationships between university researchers and the local authority, which facilitated obtaining approvals for data collection. Two cameras were mounted, and a total of 12 hours of video recordings were collected over 2 days (Table 1). Only the recordings from 1 camera were coded for analysis. The second camera acted as a backup in case there were any difficulties in coding behaviors or participant characteristics, such as instances when an object obstructed the view of the first camera. JSB coded all 12 hours of video recordings.

#### Study 3

**Figure 4:**
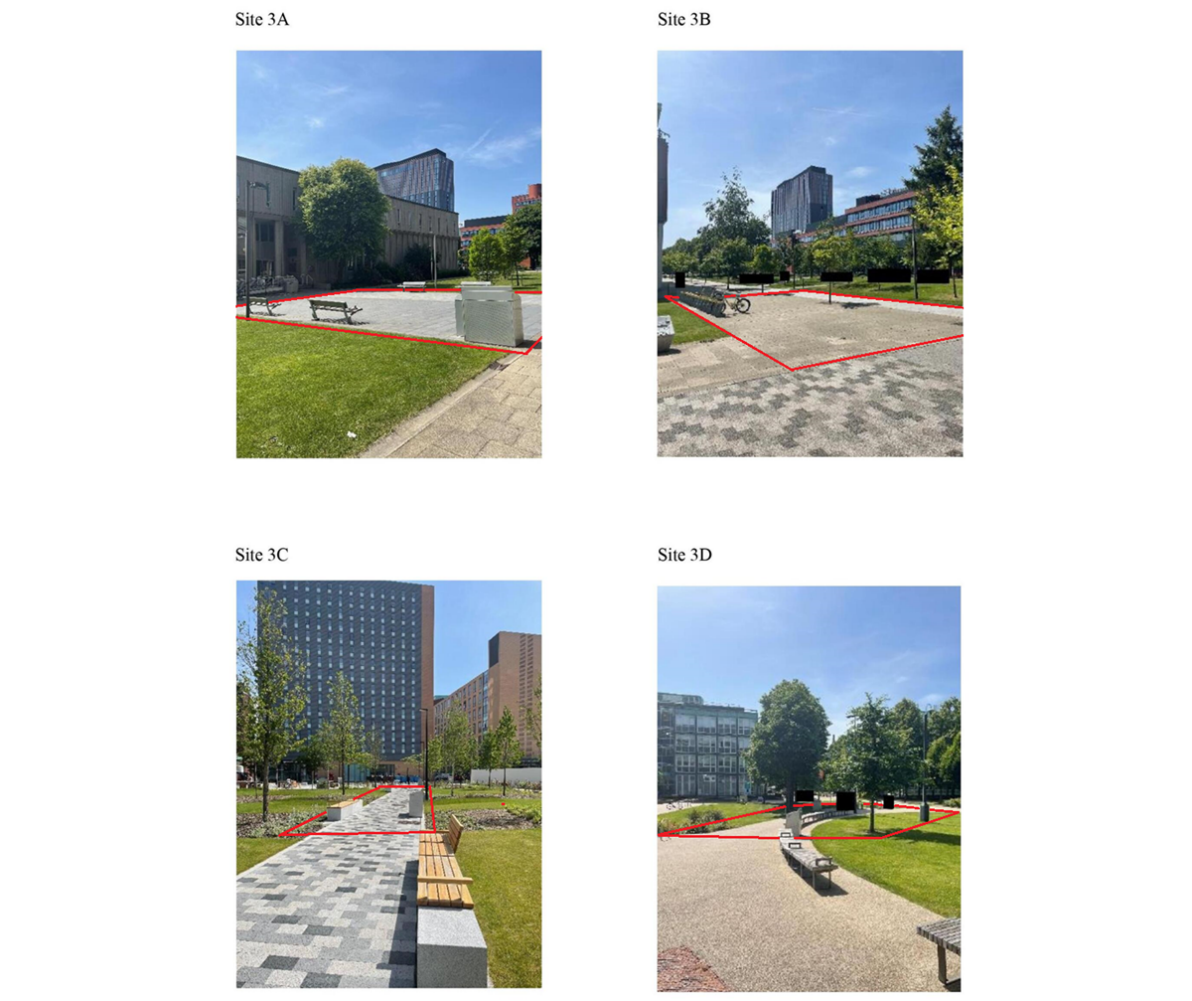
Photographs of sites 3A-3D and approximate target area boundaries (in red) for study 3. Photographs taken by JSB in June 2022. All identifying features have been masked.

**Figure 5:**
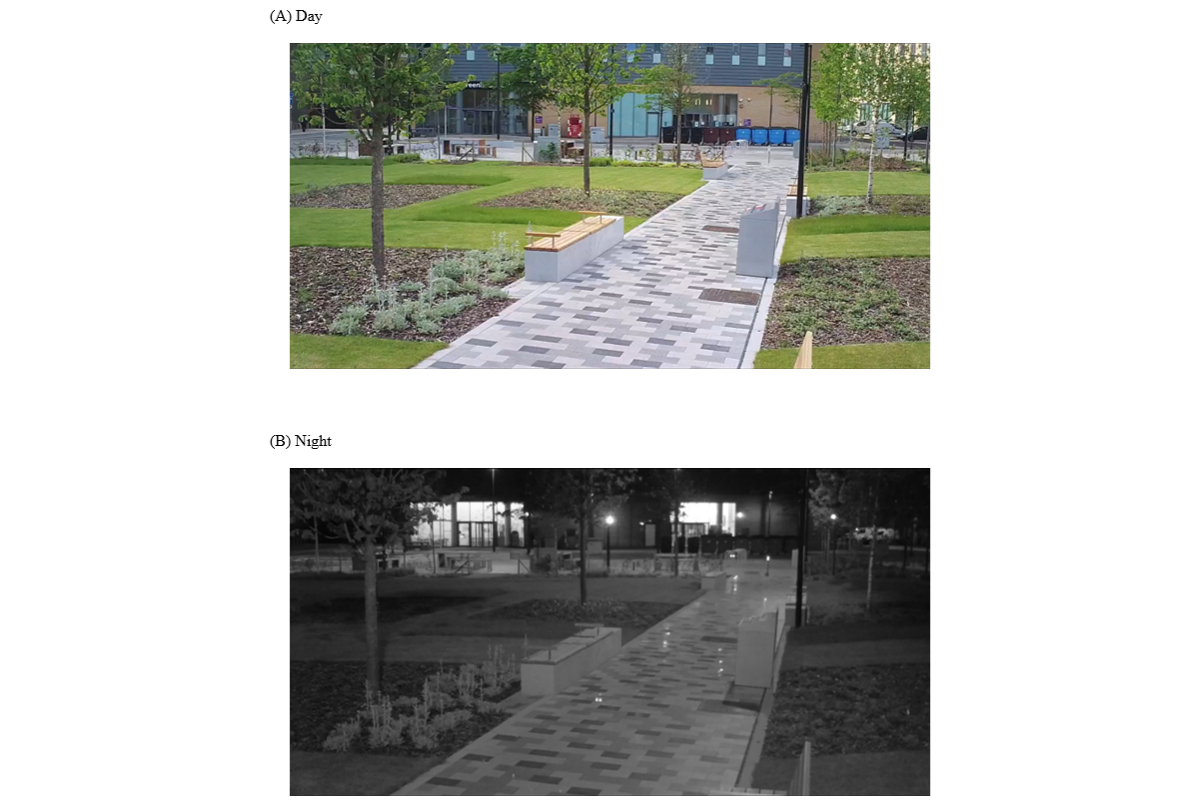
Screenshots of video recordings from site 3C during the (A) day and (B) at night for study 3. Images captured by JSB using a Reconyx XS8 UltraFire camera set to 720p image resolution.

Study 3 aimed to assess inter- and intrarater reliability, and to compare reliability between day and night observations. Two cameras were mounted at each of the 4 sites on the University of Manchester campus (Figure 4). These sites were chosen because they had adequate street lighting, which enhanced the quality of video recordings at night. A total of 20 hours of video recordings were coded per site, resulting in 80 hours of footage overall. These recordings captured various times of the day, including morning, afternoon, evening, and night (Table 1). The dataset was categorized into daytime (between 8 AM and 9 PM) and nighttime observation periods (between 10 PM and 3 AM), based on sunrise and sunset data in June for Manchester [54]. Figure 5 illustrates examples of the differences in image quality during daytime and nighttime.

For study 3, JSB (Coder 1) trained 2 additional coders (Coder 2 and Coder 3). Neither of these 2 additional coders had prior experience with MOHAWk. JSB provided approximately 10 hours of training to each coder, which included reviewing the MOHAWk instruction manual, explaining coding procedures, defining the boundaries of target areas, and coding a small sample of video recordings to resolve any discrepancies.

Coder 2 assessed all 80 hours of video recordings. To test interrater reliability, Coder 1 assessed 8 hours of these recordings, and Coder 3 assessed 20 hours. This resulted in 3 pairs of coders for interrater reliability testing—pair 1 (Coder 1 and Coder 2, 8 hours); pair 2 (Coder 1 and Coder 3, 8 hours); and pair 3 (Coder 2 and Coder 3, 20 hours). For intrarater reliability assessment, Coder 3 reassessed the same 20 hours of video recordings after a 2-week interval, but in a different randomized order to minimize potential carryover effects. Coder 3 did not receive additional training between the intervals.

#### Analyses

Inter- and intrarater reliabilities were analyzed using 2-way mixed, single measure, consistency intraclass correlation coefficients (ICCs). ICCs are appropriate for discrete data (ie, count data). Unlike Cohen κ [55], ICCs account for the magnitude of disagreement rather than all-or-nothing agreement [56]. Analyses were conducted using SPSS (version 28; IBM Corp).

### Intercept Surveys

As part of study 3, short face-to-face intercept surveys were conducted outdoors on the University of Manchester campus (Figure 4) to assess whether members of the public were aware of our video cameras and to assess whether the presence of these cameras (and surveillance cameras in general) influenced their behavior in public spaces. The surveys were conducted by JSB between 8 AM and 6 PM on Monday June 20, 2022. Although the cameras remained mounted, no video recordings were made during the survey data collection.

We used convenience sampling, aiming to recruit at least 96 English-speaking adults (aged 18 years or older, checked at introduction) at sites 3A-3D (Figure 4). For participants who agreed to take part, informed consent was obtained verbally, and all surveys were completely anonymous. Each participant was asked the questions (1) Have you noticed any cameras on the University campus today? (2) (If yes to Q1) Can you describe or point out where these cameras are? (3) (If participant identifies our cameras in Q2) Did the presence of this camera affect your behavior when passing through this area? Can you explain your answer? and (4) In general, does the presence of surveillance cameras affect your behavior in public spaces? Can you explain your answer? The estimated gender, age group, and ethnic group of each survey participant were recorded using MOHAWk coding procedures.

### Ethical Considerations

The studies were approved by the Research Ethics Committee of the University of Manchester (2020-7472-16136, 2021-10779-17985, and 2022-13577-22658). A Data Protection Impact Assessment for the processing of video recordings was approved by the University of Manchester Information Governance team in July 2020, which was a requirement under the GDPR. The need for informed consent was waived by the ethics committee. After the video data were coded and subsequently deleted, there was no risk of identifying individuals from the count data due to its low granularity. Any images used for dissemination had direct identifiable features obfuscated.

## Results

### Descriptive Overview

All 3 studies were conducted without any reported issues. A total of 3755 individuals were counted across the 3 studies (6 sites). Further descriptive summaries of counts are provided in Multimedia Appendix 2.

### Time Saved on Coding

A total of 148 hours of video recordings were coded in 85 hours. Therefore, on average, researchers took approximately 34 minutes to code each hour of video footage, which represents a time saving of 43% in coding alone. This estimate does not account for additional time savings related to travel and the intervals between observation periods, suggesting that the actual time saved is likely to be even greater.

### Interrater Reliability

Table 2 displays ICCs between each pair of coders. ICCs can be interpreted as <0.5=poor, 0.5-0.75=moderate, 0.76-0.9=good, and >0.9=excellent [57]. Interrater reliability was mostly “excellent” (n=36), with a small number of “good” (n=2), “moderate” (n=3), or “poor” (n=1) ICC values.

**Table 2:** Interrater reliability for each pair of coders.

MOHAWk^a^ code			Observer pair 1 (8 hours, 96 data points), ICC^b^ (95% CI)		Observer pair 2 (8 hours, 96 data points), ICC (95% CI)		Observer pair 3 (20 hours, 240 data points), ICC (95% CI)
**Age group**							
	Infant	N/A^c^		N/A		N/A	
	Child	1^d^ (1.00-1.00)		1^d^ (1.00 to 1.00)		1^d^ (1.00-1.00)	
	Teen	0.99^d^ (0.98-0.99)		0 (–0.2 to 0.2)		0.93^d^ (0.92-0.95)	
	Adult	0.98^d^ (0.97-0.99)		0.99^d^ (0.98 to 0.99)		0.99^d^ (0.99-0.99)	
	Older adult	1^d^ (1.00-1.00)		1^d^ (1.00 to 1.00)		0.67 (0.59-0.73)	
**Gender**							
	Female	0.97^d^ (0.95-0.98)		0.97^d^ (0.95 to 0.98)		0.99^d^ (0.98-0.99)	
	Male	0.96^d^ (0.94-0.97)		0.96^d^ (0.94 to 0.97)		0.98^d^ (0.97-0.98)	
**Ethnic group**							
	White	0.94^d^ (0.91-0.96)		0.94^d^ (0.91 to 0.96)		0.98^d^ (0.97-0.98)	
	Non-White	0.93^d^ (0.90-0.95)		0.93^d^ (0.90 to 0.96)		0.94^d^ (0.92-0.95)	
**Physical activity levels**							
	Sedentary	1^d^ (1.00-1.00)		1^d^ (1.00 to 1.00)		0.94^d^ (0.91-0.96)	
	Walking	0.99^d^ (0.98-0.99)		0.98^d^ (0.98 to 0.99)		0.97^d^ (0.96-0.97)	
	Vigorous	0.94^d^ (0.92-0.96)		0.70 (0.59 to 0.79)		0.86^e^ (0.82-0.89)	
**Well-being behaviors**							
	Connect	0.95^d^ (0.93-0.97)		0.94^d^ (0.92 to 0.96)		0.95^d^ (0.94-0.96)	
	Take notice	1^d^ (1.00-1.00)		0.80^e^ (0.71 to 0.86)		0.67 (0.59-0.73)	
**Total number of people**			0.99^d^ (0.98-0.99)		0.99^d^ (0.98 to 0.99)		0.99^d^ (0.99-0.99)

^a^MOHAWk: Method for Observing Physical Activity and Wellbeing.

^b^ICC: intraclass correlation coefficients.

^c^“N/A” means there were 0 counts.

^d^“Excellent” (ICC>0.9) reliability scores

^e^“Good” (ICC>0.75) reliability scores.

Table 3 displays a comparison of interrater reliability between day and night. Interrater reliability was generally lower at night compared to day, although there were still mostly “excellent” (n=2) or “good” (n=16) ICC values at night, with some “moderate” values (n=12). Interrater reliability reduced the most at night when coding ethnic group (White and non-White) and social interactions (connect); ICC values reduced from “excellent” to “moderate” for these 3 codes.

**Table 3:** Interrater reliability for each pair of coders at daytime and nighttime.

MOHAWk^a^ code			Observer pair 1 (8 hours, 96 data points)				Observer pair 2 (8 hours, 96 data points)				Observer pair 3 (20 hours, 240 data points)		
			Daytime, ICC^b^ (95% CI)		Nighttime, ICC (95% CI)		Daytime, ICC (95% CI)	Nighttime, ICC (95% CI)		Daytime, ICC (95% CI)			Nighttime, ICC (95% CI)
**Age group**													
	Infant	N/A^c^		N/A		N/A		N/A	N/A			N/A	
	Child	1^d^ (1.00-1.00		N/A		1^d^ (1.00-1.00)		N/A	1^d^ (1.00-1.00)			N/A	
	Teen	0.99^d^ (0.98-0.99)		N/A		0.99^d^ (0.98-0.99)		N/A	0.93^d^ (0.90-0.95)			N/A	
	Adult	0.99^d^ (0.97-0.99)		0.89^e^ (0.81-0.93)		0.99^d^ (0.97-0.99)		0.89^e^ (0.81-0.93)	0.99^d^ (0.99-0.99)			0.80^e^ (0.72-0.85)	
	Older adult	1^d^ (1.00-1.00)		N/A		1^d^ (1.00-1.00)		N/A	0.66 (0.55-0.75)			N/A	
**Gender**													
	Female	0.97^d^ (0.94-0.98)		0.89^e^ (0.81-0.94)		0.97^d^ (0.94-0.98)		0.89^e^ (0.81-0.94)	0.99^d^ (0.98-0.99)			0.78^e^ (0.70-0.84)	
	Male	0.95^d^ (0.91-0.97)		0.83^e^ (0.72-0.90)		0.95^d^ (0.91-0.97)		0.83^e^ (0.72-0.90)	0.98^d^ (0.98-0.99)			0.73 (0.64-0.81)	
**Ethnic group**													
	White	0.94^d^ (0.90-0.97)		0.69 (0.50-0.81)		0.94^d^ (0.90-0.97)		0.69 (0.50-0.81)	0.98^d^ (0.97-0.99)			0.72 (0.62-0.80)	
	Non-White	0.95^d^ (0.90-0.97)		0.66 (0.46-0.79)		0.94^d^ (0.90-0.97)		0.75 (0.60-0.85)	0.94^d^ (0.93-0.96)			0.50 (0.36-0.63)	
**Physical activity levels**													
	Sedentary	1^d^ (1.00-1.00)		1^d^ (1.00-1.00)		1^d^ (1.00-1.00)		1^d^ (1.00-1.00)	0.98^d^ (0.96-0.99)			0.66 (0.47-0.80)	
	Walking	0.99^d^ (0.98-0.99)		0.87^e^ (0.78-0.93)		0.99^d^ (0.97-0.99)		0.87^e^ (0.78-0.93)	0.96^d^ (0.94-0.97)			0.82^e^ (0.76-0.87)	
	Vigorous	0.95^d^ (0.92-0.97)		0.87^e^ (0.78-0.93)		0.62 (0.41-0.77)		0.87^e^ (0.78-0.93)	0.99^d^ (0.99-0.99)			0.56 (0.42-0.67)	
**Well-being behaviors**													
	Connect	0.98^d^ (0.97-0.99)		0.60 (0.38-0.75)		0.97^d^ (0.95-0.98)		0.60 (0.38-0.75)	0.97^d^ (0.96-0.98)			0.50 (0.35-0.62)	
	Take notice	1^d^ (1.00-1.00)		N/A		0.79^e^ (0.66-0.88)		N/A	0.67 (0.55-0.75)			N/A	
**Total number of people**			0.99^d^ (0.98-0.99)		0.89^e^ (0.81-0.93)		0.99^d^ (0.98-0.99)	0.89^e^ (0.81-0.93)		0.99^d^ (0.99-0.99)			0.80^e^ (0.72-0.85)

^a^MOHAWk: Method for Observing Physical Activity and Wellbeing.

^b^ICC: intraclass correlation coefficients.

^c^“N/A” means there were 0 counts.

^d^“Excellent” (ICC>0.9) reliability scores.

^e^“Good” (ICC>0.75) reliability scores.

### Intrarater Reliability

Table 4 displays ICCs for intrarater reliability within the same coder at 2 separate intervals. Intrarater reliability was “excellent” for all but 1 code, with 1 “good” ICC value for coding vigorous physical activity behavior.

Intrarater reliability was generally similar during the day and night, apart from ICC values for coding White and non-White (ethnic group) which reduced from “excellent” to “good” at night.

**Table 4:** Intrarater reliability at 2 separate intervals and a comparison between day and night.

MOHAWk^a^ code			All observation periods (20 hours, 240 data points), ICC^b^ (95% CI)		Daytime observation periods (10 hours, 120 data points), ICC (95% CI)		Nighttime observation periods (10 hours, 120 data points), ICC (95% CI)
**Age group**							
	Infant	N/A^c^		N/A		N/A	
	Child	1^d^ (1.00-1.00		1^d^ (1.00-1.00)		N/A	
	Teen	0.93^d^ (0.91–0.94)		0.92^d^ (0.89-0.95)		N/A	
	Adult	0.99^d^ (0.99-0.99)		0.99^d^ (0.99-0.99)		0.99^d^ (0.99-0.99)	
	Older adult	1^d^ (1.00-1.00)		1^d^ (1.00-1.00)		N/A	
**Gender**							
	Female	0.99^d^ (0.99-0.99)		0.99^d^ (0.99-0.99)		0.96^d^ (0.94-0.97)	
	Male	0.99^d^ (0.98-0.99)		0.99^d^ (0.98-0.99)		0.97^d^ (0.96-0.98)	
**Ethnic group**							
	White	0.98^d^ (0.98-0.99)		0.98^d^ (0.98-0.99)		0.78^e^ (0.69-0.84)	
	Non-White	0.96^d^ (0.94-0.97)		0.96^d^ (0.94-0.97)		0.80^e^ (0.73-0.86)	
**Physical activity**							
	Sedentary	0.97^d^ (0.96-0.98)		0.96^d^ (0.95-0.98)		1^d^ (1.00-1.00)	
	Walking	0.99^d^ (0.99-0.99)		0.99^d^ (0.99-0.99)		0.99^d^ (0.98-0.99)	
	Vigorous	0.83^e^ (0.79-0.87)		0.78^e^ (0.70-0.84)		0.94^d^ (0.92-0.96)	
**Well-being behaviors**							
	Connect	0.96^d^ (0.85-0.97)		0.96^d^ (0.94-0.97)		0.93^d^ (0.90-0.95)	
	Take notice	1^d^ (1.00-1.00)		1^d^ (1.00-1.00)		N/A	
**Total number of people**			0.99^d^ (0.99-0.99)		0.99^d^ (0.99-0.99)		0.99^d^ (0.99-0.99)

^a^MOHAWk: Method for Observing Physical Activity and Wellbeing.

^b^ICC: intraclass correlation coefficients.

^c^“N/A” means there were 0 counts.

^d^“Excellent” (ICC>0.9) reliability scores.

^e^“Good” (ICC>0.75) reliability scores.

### Intercept Surveys

A total of 86 participants completed the intercept surveys (this was 67.7% of the 127 people we approached). All participants were adults, and no older adults were included. The sample comprised 42 (48.8%) female participants and 65 (75.6%) White participants.

While 64 (74.4%) participants reported being aware of surveillance cameras on the university campus, only 5 (5.8%) participants specifically noticed the wireless video cameras used in this study. All 5 of these participants said that they did not alter their behavior in response to noticing the cameras, primarily because they expect to be recorded by surveillance cameras in outdoor public spaces on campus.

## Discussion

### Principal Findings

These 3 studies demonstrate the feasibility of using stationary wireless video cameras as a reliable and nonreactive tool for assessing physical activity and other well-being behaviors in public spaces. The use of video cameras led to a 43% reduction in coding time compared to the length of video recordings, primarily due to the ability to fast forward through periods of inactivity. Interrater reliability was mostly excellent, indicating that different observers could use the MOHAWk tool to code video recordings with high consistency. Interrater reliability was somewhat lower at night, although remained mostly excellent or good. The biggest reduction in interrater reliability at night was for coding ethnic group and social interactions. Intrarater reliability was excellent or good during the day and night, showing that individual coders could produce consistent data over a 2-week interval without additional training. Intercept surveys indicated that deploying wireless video cameras did not introduce participant reactivity.

### Comparison With Prior Work

Interrater reliability for pairs of coders (average ICC=0.92) was slightly higher than previous research using MOHAWk for in-person observations (average ICC=0.90) [11]. The improved interrater reliability with camera-based observations is likely due to 2 main factors. First, using video recordings in training makes it easier to provide clear examples of MOHAWk codes and help resolve discrepancies, rather than training with live scenes where clarifying examples may be less accessible. Second, coders can pause and rewatch footage to ensure they assign the most accurate code for each person. In contrast, in-person observers must make immediate decisions on coding, which can reduce accuracy, especially during busy times. This was shown by a previous study, which found that in-person observers counted 30% fewer walkers in a busy park compared with video coders [58]. These findings align with previous studies using in-person observations which show increased error rates as the number of observed individuals increase [17,59,60].

We found that MOHAWk can produce excellent intrarater reliability when coding video recordings after a 2-week interval. This is consistent with a previous study that found “excellent” intrarater reliability for counting individuals after a 1-week interval using drones (ICC=0.92) and “good” intrarater reliability for coding videos from a wearable video device (ICC=0.89) [61]. This suggests that camera-based methods are robust to observer drift, whereby observers become inconsistent in the criteria they use to make and record their observations over time. This is important given that observations to evaluate the effectiveness of interventions are typically conducted over several days and weeks at each time point [7]. Thus, camera-based methods can save time in future studies by avoiding too many unnecessary interrater reliability checks throughout the coding process.

Another advantage of camera-based observations compared to in-person observations is the possibility of observing at night. Conducting observations when it is dark is particularly important in winter seasons when there are typically fewer daylight hours and thus there are likely to be important differences in behaviors in outdoor environments [62]. To the authors’ knowledge, this is the first study to date that has formally tested the reliability of coding physical activity and other well-being behaviors in video recordings at night. Although interrater reliability was mostly good or excellent at night, coding ethnic group and social interactions appeared to endure the biggest reductions in interrater reliability. Improving the accuracy of assessing these codes at night will require better video technologies that can capture higher-quality color images at night. However, this must be balanced with ethical issues of intrusions to privacy from capturing higher-quality video images.

The time and flexibility afforded by camera-based observations could enhance the scalability of future natural experimental studies of environmental interventions. For example, in a recent natural experimental study where MOHAWk was used to assess an urban street greening intervention, 2 observers were used to conduct in-person observations across 11 sites (4 hour long observation periods on 2 weekdays), resulting in a total of 88 hours over 23 working days [48]. If this data collection was conducted using camera-based observations, coding would have taken approximately 50 hours over 7 working days using one coder (based on the time saved during coding in these studies). This does not include the time that would be saved on travel and the time in between observation periods. Furthermore, it would have been feasible to conduct additional observations in the evening and at weekends, providing a more comprehensive assessment of behavior.

The intercept surveys suggested that most people were not aware of our wireless video cameras. This is unsurprising given that the cameras were relatively unobtrusive in the context of these spaces (see Figure 1). Unobtrusiveness is an important strength of using camera-based observations compared with self-report measures of behavior, which are prone to measurement reactivity due to awareness of study participation [63]. Using stationary wireless cameras is also less reactive than drone-based observation methods, which have been found to cause higher reactivity by people noticing drones flying overhead [64]. The majority of survey participants reported being aware of surveillance cameras on the university campus. This is unsurprising given that the United Kingdom is one of the most surveilled countries globally [65] and thus CCTV surveillance has become normalized in many UK cities.

### Strengths and Limitations

We used both experienced and novice coders to assess video recordings at a range of times across the day and night, on weekdays and weekends, across 6 different sites. This novel research provides the foundations necessary to support further work to develop MOHAWk and other observation tools to code video recordings from stationary wireless video cameras in public spaces. We have provided extensive normative data (Multimedia Appendix 2) to further contribute to the evidence base for sample size calculations when using MOHAWk in natural experimental studies of urban environment interventions; a lack of sample size calculations is a key weakness of previous natural experimental studies of environmental interventions on physical activity [7].

However, there were some limitations to consider. We only tested cameras in sites that were purposefully selected as safe public spaces (eg, 24-hour lighting, CCTV, high footfall). This cautious approach was essential for establishing the feasibility of this relatively new research method, which is often considered high risk by university ethics committees [66]. Therefore, it is unclear whether nighttime coding would be as reliable in sites with no lighting. Additionally, the relatively small number of recording hours limited our ability to assess behavioral differences based on factors such as seasonality, holidays, and weather conditions. It is also less clear how feasible and acceptable these methods would be in public spaces that are not already being monitored by existing surveillance. It is important to note that these methods may be less suitable for public areas where individuals have higher expectations of privacy, such as near schools or hospitals.

Coders were trained to avoid double counting of the same individual within each hour-long observation period, as specified by MOHAWk procedures. However, we did not formally assess whether coders avoided double counting, which can be a concern in systematic observation. The potential for double counting might have contributed to the lower interrater reliability for coding vigorous physical activity, particularly in the case of large groups of teenagers observed on bikes during afternoon sessions. Despite this, the high interrater reliability for counting the total number of people (as shown in Table 2) indicates that the incidence of double counting in this study was likely minimal.

The intercept survey sample size (n=86) fell short of the target of 96 participants, as calculated a priori. Also, surveys were limited to sites on the university campus, resulting in a sample predominantly composed of university students and staff. This was due to time and resource limitations in conducting surveys while the cameras were still mounted. Larger-scale survey research is needed to make more generalizable inferences for different population groups and different types of public spaces, particularly those with fewer existing surveillance cameras. Further qualitative research is also needed to explore the acceptability of camera-based methods, providing insights into privacy expectations across different types of public spaces, locations, and cultures.

### Implications

We have compiled a nonexhaustive list of considerations for researchers, based on our experiences using stationary wireless video cameras in public spaces (Textbox 1). This guidance is intended to help researchers develop camera-based observation methods in a reliable and responsible manner, thereby unlocking the considerable potential of camera-based research across health and social sciences. It is essential that researchers take the necessary steps to ensure that video cameras are used legally, ethically, and responsibly within their research setting, addressing issues concerning data protection, privacy, consent, and confidentiality. We have offered a detailed discussion elsewhere of how to use video cameras ethically and in line with stringent data protection legislation under the GDPR [26].

There is a scarcity of robust natural experimental studies of the causal effects of urban environment interventions on physical activity [7,67]. This research highlights that camera-based observation methods have good psychometric properties and have the potential to reduce the time and cost needed to generate more robust intervention-based evidence. This is important as there is increasing demand for high-quality, practice-based evidence in policy making [68], driven by the growing number of local and national policies in the United Kingdom and worldwide that recommend improving urban environments to promote physical activity [7]. By combining camera-based observations with other methods (eg, self-report, accelerometers, and sensors) within natural experimental research, we can ensure that findings are robust to the different types of bias inherent in each individual method of measurement.

Camera-based observation methods can be used to assess a wider range of health and well-being behaviors that are observable and relevant to public space usage. For example, previous research has used in-person observations to study smoking behavior in order to evaluate the effectiveness of a policy intervention to ban smoking in enclosed public spaces [69]. Hence, camera-based methods could be used to provide valuable data for important health and policy-related questions where robust data are currently lacking due to an overreliance on traditional survey-based measures.

**Considerations for using stationary wireless video cameras as a research tool in public spaces.**

**Identifying a suitable camera**

- Ensure the camera has adequate image resolution to support reliable coding.
- Use cameras with infrared night vision capabilities if nighttime coding is required.
- Check that the camera has sufficient memory capacity and battery life for extended recording periods.
- Recording videos during set intervals is more reliable than relying on motion detection but will consume more battery life.
- Avoid excessive zoom functionalities that do not contribute to the research objectives.
- Prioritize security features (eg, password protection and encryption) and store video recordings locally, rather than on a cloud, to minimize risks of hacking.
- Select cameras that are resistant to harsh weather conditions, including rain and extreme temperatures.
- Ensure the camera be securely mounted on flat vertical surfaces (eg, lamp posts).

**Prior to camera installation**

- Obtain permission from landowners before installing any cameras.
- Engage with relevant stakeholders, including members of the public who are likely to be recorded.
- Develop clear policies regarding the use and storage of video recordings, including predefined procedures for reporting any illegal activities captured on video.
- Display multiple privacy notice signs before data collection begins (eg, 1 week in advance) to notify regular users of the study site.
- Privacy notices should direct individuals to more detailed privacy information via a separate participant information sheet, which should be accessible both physically at the study site (eg, printed and in a laminate folder) and web-based (eg, via a QR code or website address).

**Camera installation**

- Use at least 2 cameras at each site, especially in areas where objects might obstruct the camera’s view, or if detailed demographic coding is necessary.
- Carefully consider the height and angle of cameras to optimize coverage of the scene.
- Account for objects that could block the field of view, such as large vehicles.
- Mount cameras at a height that minimizes the risk of tampering or theft.
- Use secure screw mounts, ideally placing cameras inside a padlocked security case, to ensure they remain fixed throughout the study.
- Conduct a preliminary phase of video recording to verify that images are clear and that the positioning of the cameras is appropriate.
- Perform any necessary maintenance checks, including replacing batteries and memory cards.

**Coding video recordings**

- Establish debriefing and support processes for coders who may witness distressing footage.
- Ensure that coders are adequately trained and fully understand all data protection procedures.
- Implement interrater reliability tests among coders using a sample of video recordings and resolve discrepancies to achieve at least “good” agreement (ICCs>0.75) across all codes.
- Use digital platforms (eg, tablets) during the coding process to facilitate streamlined data entry and analysis.

### Future Research

Further psychometric testing is needed to establish the reliability of camera-based observation methods in urban green spaces (eg, parks, greenways, and canals), where many natural experimental studies on physical activity are commonly conducted [4,70]. Given that these studies were conducted under favorable weather conditions, reliability testing is also needed in varied weather conditions, such as rain or fog, which may impact coding accuracy. Moreover, video cameras could also be used to validate the MOHAWk observation tool. For example, by conducting intercept surveys to compare raters’ estimated demographic characteristics and participants’ self-reported demographic information.

This research used 1 type of camera and did not explore the effects of different camera mounting positions. Since capturing clear images is crucial for reliable behavioral assessment, further studies should explore how different camera types and mounting positions influence data reliability. For example, mounting cameras at higher positions could reduce the likelihood of behaviors being obscured by people in busy public spaces.

Despite the advantages of using camera-based observations over traditional in-person observation methods, the approach used in these studies still requires significant human labor. As a result, data collection is limited to short time periods and a small number of sites. This issue of scalability across time and space could be addressed by developing automated observation methods. Researchers are beginning to capitalize on advances in machine learning technology within the field of computer vision to accurately recognize and track humans in video images. For example, deep learning models have been tested to estimate the number of people in a park and to determine how many are engaged in physical activity using fixed video cameras [40], as well as to assess physical activity intensities with participant-worn cameras [71]. Developing automated methods could enable rapid and long-term monitoring to inform environmental interventions and might provide greater consistency compared to manual human coding. Furthermore, if these automated methods can be developed in a data-secure manner, they may offer enhanced privacy by eliminating the need for humans to view video footage. However, many questions remain regarding the development of deep learning models, particularly concerning issues of privacy, accuracy, and generalizability. Exploring automated technologies has been identified as a top priority in the field of physical activity observation research [40].

### Conclusions

The results from 148 hours of camera-based observations, conducted by 3 coders across 6 different urban spaces, indicate that using video cameras is a feasible, reliable, and nonreactive method for assessing physical activity and other well-being behaviors in public spaces. These findings show that camera-based observation methods are more reliable than in-person observation methods and do not produce participant reactivity typically associated with self-report. This method requires less time for data collection and coding, while allowing for safe nighttime observation without risk to research staff. This research is a crucial first step in demonstrating the potential for camera-based methods to improve and scale up natural experimental studies of real-world environmental interventions. It also provides an important foundation for developing more scalable automated computer vision algorithms for assessing human behaviors.

## Appendix

Multimedia Appendix 1 Completed STROBE (Strengthening the Reporting of Observational Studies in Epidemiology) checklist.

Multimedia Appendix 2 Mean counts of participant characteristics and well-being behaviors per hour for all 3 studies (6 sites).

## Abbreviations

CCTV: closed-circuit television
GDPR: General Data Protection Regulation
ICC: intraclass correlation coefficient
MOHAWk: Method for Observing Physical Activity and Wellbeing
SOPARC: System for Observing Play and Active Recreation in Communities
STROBE: Strengthening the Reporting of Observational Studies in Epidemiology

## Data Availability

The datasets generated and analyzed during this study are available from the corresponding author on reasonable request.
