# Supplementary material for: Using Video Cameras to Assess Physical Activity and Other Well-Being Behaviors in Urban Environments: Feasibility, Reliability, and Participant Reactivity Studies
Source: JMIR Public Health Surveill. 2024 Dec 16;10:e66049. doi: 10.2196/66049 (PMC11686020; doi:10.2196/66049)
Supplement: Multimedia Appendix 1 [file publichealth_v10i1e66049_app1.docx]

This checklist was elaborated using formal items recommended for cross-sectional studies from STROBE statement (https://www.strobe-statement.org).

|  | Item No | Recommendation | Respected? | Comments and quotes |
| --- | --- | --- | --- | --- |
| **Title and abstract** | 1 | (*a*) Indicate the study’s design with a commonly used term in the title or the abstract | Yes | Study design is indicated in the methods section of the abstract:  “Across three cross-sectional studies, 148 hours of video recordings were collected from six outdoor public spaces in Manchester, UK” |
|  |  | (*b*) Provide in the abstract an informative and balanced summary of what was done and what was found | Yes | This information is stated in the study abstract (study objective described, method and results described) |
| Introduction | | |  |  |
| Background/rationale | 2 | Explain the scientific background and rationale for the investigation being reported | Yes | Rationale and existing literature are stated in the introduction section |
| Objectives | 3 | State specific objectives, including any prespecified hypotheses | Yes | A statement at the end of the introduction specifies the aims and specific objectives.  “To address these methodological concerns, this article reports three studies that aimed to test the feasibility of conducting reliable and non-reactive systematic observation of physical activity and other wellbeing behaviours by coding video recordings collected from stationary wireless video cameras. Specific objectives of these three studies were to: (1) test the feasibility of deploying wireless video cameras as a research tool in public spaces (Studies 1, 2, and 3); (2) assess inter-rater reliability between pairs of coders (Study 3); (3) assess intra-rater reliability within a single coder at two separate intervals (Study 3); (4) compare inter- and intra-rater reliability between day and night (Study 3); and (5) use intercept surveys to examine public space users’ awareness of and reactivity to cameras (Study 3).” |
| Methods | | |  |  |
| Study design | 4 | Present key elements of study design early in the paper | Yes | Study design is stated in the first subsection of Methods. Key elements are described in the methods.  “There were three prospective cross-sectional studies. Each study had two phases of camera-based data collection. The first phase involved ensuring that video recordings were taken without objections from members of the public, test the clarity of images, and determine the optimal positioning of cameras. Any adjustments were made before proceeding to the second phase of data collection that was used for coding. In the third study, intercept surveys were also conducted with public space users to examine their awareness of and reactivity to our cameras.” |
| Setting | 5 | Describe the setting, locations, and relevant dates, including periods of recruitment, exposure, follow-up, and data collection | Yes | Setting, contexts, relevant dates, are described in the methods section under “Setting”. |
| Participants | 6 | (*a*) Give the eligibility criteria, and the sources and methods of selection of participants | Yes | Study population is described in the methods section (under “Procedures”), as well as selection criteria:  “Coders used the MOHAWk tool to assess the following estimated information for each person that entered a pre-determined target area during pre-specified hour-long observation periods: gender (Female or Male), age group (Infant, Child, Teen, Adult or Older Adult), ethnic group (White or Non-white), physical activity level (Sedentary, Walking, Vigorous), social interaction (Connect or No Connect), and taking notice of the environment (Take Notice or No Take Notice).”  Eligibility criteria and method of selection of intercept survey participants is described in the methods section (under “Intercept surveys”):  “We used convenience sampling, aiming to recruit at least 96 English-speaking adults (aged 18+ checked in introduction) at Sites 3A – 3D (Figure 4).” |
| Variables | 7 | Clearly define all outcomes, exposures, predictors, potential confounders, and effect modifiers. Give diagnostic criteria, if applicable | Yes | Standardised variable definitions were used based on the MOHAWk tool, which are presented in methods section. As these studies focused on testing psychometrics of the tool itself, there were no potential confounders. The questions for the intercept survey are provided in the methods under “Intercept surveys”:  “Each participant was asked the following questions:   1. Have you noticed any cameras on the University campus today? 2. [if yes to Q1] Can you describe or point out where these cameras are? 3. [if participant identifies our cameras in Q2] Did the presence of this camera affect your behaviour when passing through this area? Can you explain your answer? 4. In general, does the presence of surveillance cameras affect your behaviour in public spaces? Can you explain your answer?” |
| Data sources/ measurement | 8* | For each variable of interest, give sources of data and details of methods of assessment (measurement). Describe comparability of assessment methods if there is more than one group | Yes | Data collection and measurement was the same for all variables of the MOHAWk tool and is described in the methods section. Data assessment methods are described in detail under the “Procedures” subsection in the methods. |
| Bias | 9 | Describe any efforts to address potential sources of bias | Yes | In the methods (under “Study design”), we describe efforts to ensure the camera positioning reduced risk of missing data:  “The first phase involved ensuring that video recordings were taken without objections from members of the public, test the clarity of images, and determine the optimal positioning of cameras. Any adjustments were made before proceeding to the second phase of data collection that was used for coding”  In the “Strengths and limitations” sub-section in the discussion, we reflect on potential limitations in the sampling of locations and times, as well as issues associated with double counting:  “However, there were some limitations to consider. We only tested cameras in sites that were purposefully selected as safe public spaces (e.g., 24-hour lighting, CCTV, high footfall). This cautious approach was essential for establishing the feasibility of this relatively new research method, which is often considered high risk by university ethics committees ^65^. Therefore, it is unclear whether night-time coding would be as reliable in sites with no lighting. Additionally, the relatively small number of recording hours limited our ability to assess behavioural differences based on factors such as seasonality, holidays, and weather conditions. It is also less clear how feasible and acceptable these methods would be in public spaces that are not already being monitored by existing surveillance. It is important to note that these methods may be less suitable for public areas where individuals have higher expectations of privacy, such as near schools or hospitals. CCoders were trained to avoid double counting of the same individual within each hour-long observation period, as specified by MOHAWk procedures. However, we did not formally assess whether coders avoided double counting, which can be a concern in systematic observation. The potential for double counting might have contributed to the lower inter-rater reliability for coding vigorous physical activity, particularly in the case of large groups of teenagers observed on bikes during afternoon sessions. Despite this, the high inter-rater reliability for counting the total number of people (as shown in Table 2) indicates that the incidence of double counting in this study was likely minimal.”  Sample size issues for the intercept surveys are reflected in the “Strengths and limitations” subsection in the discussion:  “The intercept survey sample size (*n* = 86) fell short of the target of 96 participants, as calculated a priori. Also, surveys were limited to sites on the university campus, resulting in a sample predominantly composed of university students and staff. This was due to time and resource limitations in conducting surveys while the cameras were still mounted. Larger-scale survey research is needed to make more generalisable inferences for different population groups and different types of public spaces, particularly those with fewer existing surveillance cameras.” |
| Study size | 10 | Explain how the study size was arrived at | Partially | The methods section describes justification for the observation scheduling:  “Previous research has shown that shortened observation schedules (e.g., two days, four times a day) can yield reliable estimates of activity in a public space”  We described the desired target sample size for the intercept surveys in the discussion, although we did not explain the details of the calculation:  “The intercept survey sample size (*n* = 86) fell short of the target of 96 participants, as calculated a priori.” |
| Quantitative variables | 11 | Explain how quantitative variables were handled in the analyses. If applicable, describe which groupings were chosen and why | Yes | We explained how we compared daytime vs. night-time observation periods in the methods section (under “Study 3”):  “The dataset was categorised into daytime (between 8am and 9pm) and night-time observation periods (between 10pm and 3am), based on sunrise and sunset data in June for Manchester” |
| Statistical methods | 12 | (*a*) Describe all statistical methods, including those used to control for confounding | Yes | These are described in the methods section.  “Inter- and intra-rater reliabilities were analysed using two-way mixed, single measure, consistency ICCs. ICCs are appropriate for discrete data (i.e., count data). Unlike Cohen’s kappa ^55^, ICCs account for the magnitude of disagreement rather than all-or-nothing agreement ^56^. Analyses were conducted using SPSS version 28.” |
|  |  | (*b*) Describe any methods used to examine subgroups and interactions | Yes | This is described in the methods section for daytime vs. night-time observations. Interaction examination was not applicable. |
|  |  | (*c*) Explain how missing data were addressed | N/A | N/A |
|  |  | (*d*) If applicable, describe analytical methods taking account of sampling strategy | N/A | N/A |
|  |  | (*e*) Describe any sensitivity analyses | N/A | N/A |
| Results | | |  |  |
| Participants | 13* | (a) Report numbers of individuals at each stage of study—eg numbers potentially eligible, examined for eligibility, confirmed eligible, included in the study, completing follow-up, and analysed | Yes | This is described at the beginning of the results section and in Multimedia Appendix 2:  “A total of 3,755 individuals were counted across the three studies (six sites). Further descriptive summaries of counts are provided in Multimedia Appendix 2.”  The sample size for intercept surveys is also described in the results:  “A total of 86 participants completed the intercept surveys” |
|  |  | (b) Give reasons for non-participation at each stage | No | This is not applicable to the observations.  For the intercept surveys, although the number of participants who agreed to take part was reported (67.7%), the reasons for non-participation were not collected, and thus were not described. |
|  |  | (c) Consider use of a flow diagram | N/A | Use of a flow diagram was not deemed appropriate |
| Descriptive data | 14* | (a) Give characteristics of study participants (eg demographic, clinical, social) and information on exposures and potential confounders | Yes | Multimedia Appendix 2 describes the observed characteristics of participants for the observations.  Observed characteristics of the intercept survey participants are described in the results section:  “All participants were adults, and no older adults were included. The sample comprised 42 females (48.8%) and 65 white participants (75.6%).” |
|  |  | (b) Indicate number of participants with missing data for each variable of interest | N/A | N/A |
| Outcome data | 15* | Report numbers of outcome events or summary measures | Yes | All numbers for the observations are reported in Tables and Multimedia Appendix 2.  Summary of intercept survey responses are described in the results section:  “While 64 participants (74.4%) reported being aware of surveillance cameras on the university campus, only five participants (5.8%) specifically noticed the wireless video cameras used in this study. All five of these participants said that they did not alter their behaviour in response to noticing the cameras, primarily because they expect to be recorded by surveillance cameras in outdoor public spaces on campus.” |
| Main results | 16 | (*a*) Give unadjusted estimates and, if applicable, confounder-adjusted estimates and their precision (eg, 95% confidence interval). Make clear which confounders were adjusted for and why they were included | Yes | All unadjusted estimates, and 95% confidence intervals, are reported in Tables 2-4. There were no adjustment estimates or confounders. |
|  |  | (*b*) Report category boundaries when continuous variables were categorized | N/A | N/A |
|  |  | (*c*) If relevant, consider translating estimates of relative risk into absolute risk for a meaningful time period | N/A | N/A |
| Other analyses | 17 | Report other analyses done—eg analyses of subgroups and interactions, and sensitivity analyses | N/A | N/A |
| Discussion | | |  |  |
| Key results | 18 | Summarise key results with reference to study objectives | Yes | Key results are described at the beginning of discussion section. They are also summarised in the conclusion. |
| Limitations | 19 | Discuss limitations of the study, taking into account sources of potential bias or imprecision. Discuss both direction and magnitude of any potential bias | Yes | Description of limitations is done under “Strengths and limitations” heading in the discussion. |
| Interpretation | 20 | Give a cautious overall interpretation of results considering objectives, limitations, multiplicity of analyses, results from similar studies, and other relevant evidence | Yes | References were added where possible and discussed. Limitations were considered in the discussion. |
| Generalisability | 21 | Discuss the generalisability (external validity) of the study results | Yes | Issues of generalisability were discussed under “Strengths and limitations” heading in the discussion. |
| Other information | | |  |  |
| Funding | 22 | Give the source of funding and the role of the funders for the present study and, if applicable, for the original study on which the present article is based | Yes | Funding information were described under “Acknowledgements”. |

*Give information separately for exposed and unexposed groups.
