# Supplementary material for: Using Video Cameras to Assess Physical Activity and Other Well-Being Behaviors in Urban Environments: Feasibility, Reliability, and Participant Reactivity Studies
Source: JMIR Public Health Surveill. 2024 Dec 16;10:e66049. doi: 10.2196/66049 (PMC11686020; doi:10.2196/66049)
Supplement: Multimedia Appendix 2 [file publichealth_v10i1e66049_app2.docx]

| **Study** | **Site** | **Total hours** | **Mean count per hour (standard deviation) and total count, *n***^a^ | | | | | | | | | | | | | | |
| --- | --- | --- | --- | --- | --- | --- | --- | --- | --- | --- | --- | --- | --- | --- | --- | --- | --- |
|  |  |  | **Age group** | | | | | **Gender** | | **Ethnic group** | | **Physical activity levels** | | | **Wellbeing behaviours** | | **Total number of people** |
|  |  |  | **Infant** | **Child** | **Teen** | **Adult** | **Older Adult** | **Female** | **Male** | **White** | **Non-White** | **Sedentary** | **Walking** | **Vigorous** | **Connect** | **Take notice** |  |
| Study 1^b^ | Site 1 | 8 hours | 0.13  (0.35)  *n = 1* | 0  (0)  *n = 0* | 0.25  (0.71)  *n = 2* | 29.70  (11.19)  *n = 238* | 0  (0)  *n = 0* | 15.38  (6.67)  *n = 123* | 14.58  (5.77)  *n = 117* | 21.73  (9.75)  *n = 174* | 8.35  (3.64)  *n = 65* | 0.50  (1.07)  *n = 4* | 28.38  (9.23)  *n = 227* | 2.75  (3.88)  *n = 22* | 11.40  (8.04)  *n = 91* | 1.10  (2.73)  *n = 9* | 30.08  (11.65)  *n = 241* |
| Study 2^b^ | Site 2 | 12 hours | 4.67  (4.36)  *n = 56* | 18  (22.32)  *n = 216* | 21.83  (22.57)  *n = 262* | 55.33  (23.92)  *n = 664* | 3.67  (2.57)  *n = 44* | 41.83  (29.73)  *n = 502* | 57  (32.56)  *n = 684* | 72.08  (45.65)  *n = 865* | 26.75  (17.26)  *n = 321* | 1.5  (1.78)  *n = 18* | 82.33  (48.32)  *n = 988* | 17.17  (15.19)  *n = 206* | 39.92  (27.80)  *n = 479* | 1.25  (1.48)  *n = 15* | 103.5  (65.26)  *n = 1,242* |
| Study 3^c^ | Site 3A | 20 hours | 0.10 (0.45)  *n = 2* | 0.10 (0.45)  *n = 2* | 1.20  (3.29)  *n = 24* | 29.75 (43.89)  *n = 595* | 0.30  (0.80)  *n = 6* | 15.70  (23.93)  *n = 314* | 15.65  (22.67)  *n = 313* | 19.20  (30.52)  *n = 384* | 12.15  (16.07)  *n = 243* | 3.40  (7.26)  *n = 68* | 30.35  (44.31)  *n = 607* | 0.90  (1.68)  *n = 18* | 11.60  (21.50)  *n = 232* | 0.05  (0.22)  *n = 1* | 31.45  (46.02)  *n = 629* |
|  | Site 3B | 20 hours | 0.10 (0.45)  *n = 2* | 0.75 (1.29)  *n = 15* | 5.65 (15.57)  *n = 113* | 83.55 (88.13)  *n = 1,671* | 0.65  (1.31)  *n = 13* | 41.50  (47.74)  *n = 830* | 49.10  (47.05)  *n = 982* | 53.40  (62.39)  *n = 1,068* | 37.20  (34.00)  *n = 744* | 0.25  (0.72)  *n = 5* | 82.05  (88.66)  *n = 1,641* | 9.05  (7.85)  *n = 181* | 29.95  (30.36)  *n = 599* | 0.25  (0.55)  *n = 5* | 90.70  (94.46)  *n = 1,814* |
|  | Site 3C | 20 hours | 0  (0)  *n = 0* | 0  (0)  *n = 0* | 0.10  (0.45)  *n = 2* | 17.85  (19.27)  *n = 357* | 0.10  (0.31)  *n = 2* | 7.70  (8.99)  *n = 154* | 10.35  (12.18)  *n = 207* | 12.85  (16.26)  *n = 257* | 5.20  (4.48)  *n = 104* | 1.55  (3.79)  *n = 31* | 16.45  (18.97)  *n = 329* | 1.50  (1.54)  *n = 30* | 5.15  (6.34)  *n = 103* | 0.10  (0.45)  *n = 2* | 18.05  (19.57)  *n = 361* |
|  | Site 3D | 20 hours | 0  (0)  *n = 0* | 0.15  (0.15)  *n = 3* | 1.80  (7.82)  *n = 36* | 13.80  (20.26)  *n = 276* | 0.20  (0.62)  *n = 4* | 8.00  (13.27)  *n = 160* | 7.95  (12.31)  *n = 159* | 10.30  (17.99)  *n = 206* | 5.65  (9.21)  *n = 113* | 4.60  (9.73)  *n = 92* | 15.00  (25.06)  *n = 300* | 1.00  (1.12)  *n = 20* | 7.80  (14.92)  *n = 156* | 0  (0)  *n = 0* | 15.95  (25.41)  *n = 319* |

^a^Each observed person can engage in more than one behaviour per observation period e.g., a person who is walking and talking to a friend would be coded as Walking’ and ‘Connect’.

^b^Data coded by JSB.

^c^Data coded by Coder 2.
